# Exploring the Dynamics of Oligodendrocyte Precursor Cell Differentiation in Three-dimensional Culture Systems

**DOI:** 10.14789/jmj.JMJ23-0046-OT

**Published:** 2024-06-15

**Authors:** HINATA NISHIMURA

**Affiliations:** 1 Research Institute for Diseases of Old Age, Juntendo University Graduate School of Medicine, Tokyo, Japan; 1 Research Institute for Diseases of Old Age, Juntendo University Graduate School of Medicine, Tokyo, Japan

**Keywords:** chondroitin sulfate, extracellular matrix (ECM), oligodendrocytes, oligodendrocyte precursor cell (OPC), decellularized tissue

The goal of this study is to investigate the interactions between chondroitin sulfate (CS) chains and oligodendrocyte lineage cells (Figure 1a). Conventional two-dimensional (2D) culture systems do not adequately mimic the complex structure of the extracellular matrix (ECM) or simulate in vivo conditions. We previously developed a three-dimensional (3D) culture system based on a decellularized brain tissue1) and tested it using the oligodendrocyte cell line OL6^1)^. As the next step, the system was validated using primary cultures of oligodendroglial lineage. To evaluate neural stem cell differentiation in the 3D culture system, embryonic neurons were transplanted into the decellularized tissue; however, this approach was not successful because of the predominant differentiation of the neural stem cells into astrocytes. To enhance the differentiation of oligodendroglia, the standard protocol was optimized and cells were cultured in media supplemented with hepatocyte growth factor (HGF) and platelet-derived growth factor AA (PDGFAA). The schematic representation of this protocol is summarized in Figures 1b,c. One week after transplantation, the presence of oligodendrocyte precursor cells (OPCs), immature oligodendrocytes (iOL I), and mature oligodendrocytes was confirmed by immunostaining using differentiation-specific markers. A comparison between 2D and 3D cultures systems demonstrated that 3D conditions effectively induced the differentiation of neural stem cells into oligodendrocyte lineages (manuscript in preparation). The CS56 antibody staining results showed that the signal was stronger in 3D cultures compared to that in 2D cultures. These findings indicate that the 3D culture system provides optimal conditions for investigating the crosstalk between OPC differentiation and the ECM structure.

**Figure 1:**
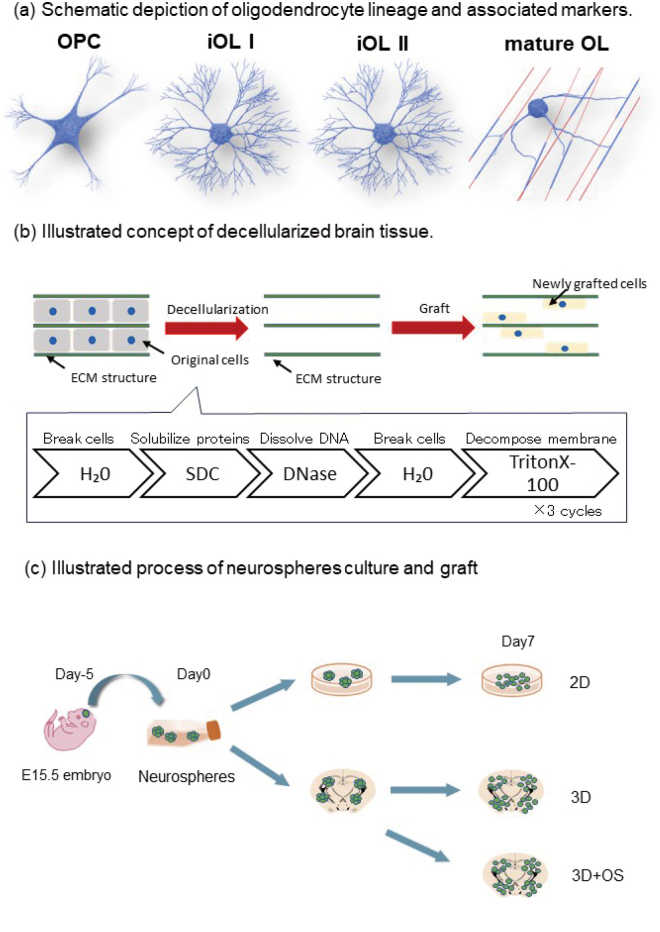
(a) Schematic representation of the oligodendrocyte lineage and its associated markers. (b) The diagram of decellularized brain tissue for grafting. Post-grafting, both 2D and 3D samples were cultured in a differentiation medium supplemented with hepatocyte growth factor (HGF). The “3D+OS” samples were cultured in an oligosphere medium containing both HGF and platelet-derived growth factor AA (PDGFAA).

## Funding

No funding was received.

## Author contributions

The author read and approved the final manuscript.

## Conflicts of interest statement

The author declares that there are no conflicts of interest.
